# Semisupervised hyperspectral image classification based on generative adversarial networks and spectral angle distance

**DOI:** 10.1038/s41598-023-49239-2

**Published:** 2023-12-12

**Authors:** Ying Zhan, Yufeng Wang, Xianchuan Yu

**Affiliations:** 1 School of Computer and Software, Nanyang Institute of Technology, Nanyang, 473000 China; 2 School of Artificial Intelligence, Beijing Normal University, Beijing, 100875 China

**Keywords:** Geology, Computer science

## Abstract

Collecting ground truth labels for hyperspectral image classification is difficult and time-consuming. Without an adequate number of training samples, hyperspectral image (HSI) classification is a challenging problem. Using generative adversarial networks (GANs) is a promising technique for solving this problem because GANs can learn features from both labeled and unlabeled samples. The cost functions widely used in current GAN methods are suitable for 2D nature images. Compared with natural images, HSIs have a simpler one-dimensional structure that facilitates image generation. Motivated by the one-dimensional spectral features of HSIs, we propose a novel semisupervised algorithm for HSI classification by introducing spectral angle distance (SAD) as a loss function and employing multilayer feature fusion. Since the differences between spectra can be quickly calculated using the spectral angle distance, the convergence speed of the GAN can be improved, and the samples generated by the generator model in the GAN are closer to the real spectrum. Once the entire GAN model has been trained, the discriminator can extract multiscale features of labeled and unlabeled samples. The classifier is then trained for HSI classification using the multilayer features extracted from a few labeled samples by the discriminator. The proposed method was validated on four hyperspectral datasets: Pavia University, Indiana Pines, Salinas, and Tianshan. The experimental results show that the proposed model provides very promising results compared with other related state-of-the-art methods.

## Introduction

The hyperspectral images (HSIs) acquired by hyperspectral sensors can simultaneously contain hundreds of continuous narrow spectral bands and spatial information. With such rich information, HSIs can be widely applied in many areas, such as land cover/use classification and recognition^1,2^, water pollution detection, and mineral exploration. In these applications, the classification of each pixel in the HSI plays a vital role. To improve the accuracy of HSI classification, many classification methods have been developed for remote sensing applications.

The current classification methods can be divided into three categories: unsupervised, supervised, and semisupervised learning^3^. Unsupervised learning methods, such as graph-based methods^4^, artificial DNA computing^5^, and fuzzy-based methods^6^, do not require labeled samples and can quickly cluster samples. However, the classification accuracy of unsupervised methods is usually lower than that of supervised methods, and it is difficult to judge the number of classes and guarantee a relationship between the clusters and classes.

By utilizing a priori information of the class labels, supervised classifiers can show improved performance and are thus widely applied in remote sensing image processing. A characteristic of supervised classifiers is that they can be used to distinguish between several classes. Typical supervised classifiers include the maximum likelihood classifier (MLC)^7,8^, support vector machines (SVMs)^9,10^, and convolutional neural networks (CNNs)^11,12^.

However, due to the large number of spectral bands in HSIs, a highly accurate supervised classification model requires many training samples. On the one hand, collecting labeled samples is difficult and expensive; on the other hand, the overall features of HSIs are difficult to obtain from small sets of labeled samples, which will lead to the problem of model underfitting.

Semisupervised learning (SSL) can alleviate the above problems because this approach can obtain features from both unlabeled samples and labeled samples^13^. Existing SSL methods can be divided into generative model methods and discriminative model methods. Generative model methods, such as the Markov random field^14^ and soft sparse multinomial logistic regression^15^, attempt to model the real data distribution directly. Discriminative model methods directly group the data into well-separated categories using certain classification methods, such as graph-based methods^16–18^ and wrapper-based methods^19,20^.

Moreover, most of the methods can classify HSIs in only a “shallow” manner. Compared with “shallow” methods, deep methods can obtain more features from the training samples and therefore have more advantages when handling high-dimensional data. To date, many deep learning methods, especially deep convolutional neural networks (CNNs), have been successfully utilized for image processing^21^ and rapidly applied for the classification of HSIs^22^.

At present, a series of deep learning methods based on generative adversarial networks (GANs) have been successfully applied in image classification and recognition^23,24^. GANs were first introduced by Goodfellow^25^. These networks combine a generative model with a discriminative model. The generative model $G$ tries to generate a distribution that is as similar as possible to that of the real data, and the discriminative model $D$ determines whether the samples are from the real data or $G$. From the perspective of the generator, this is a data augmentation method; from the perspective of the discriminator, the data features can be learned while training the GAN using the unlabeled data. Therefore, GANs are suitable for semisupervised learning (SSL). Currently, a series of SSL and unsupervised learning methods based on the GAN are rapidly being developed^26^. For example, the categorical GAN^27^ can handle image classification tasks as well as generate data, making it a method that can learn a discriminative classifier from unlabeled or partially labeled data. GANs can also perform semisupervised classification by forcing the discriminator network to output class labels^28^. GANs have also been applied in the field of remote sensing image classification^29^. Spectral spatial features were extracted by 3DBF using a semisupervised method based on a GAN^30^. Later, a novel GAN-based method^31^ was proposed as a HSIs classifier, which achieved a good level of performance with a limited number of labeled samples. Previously, HSGANs (hyperspectral generative adversarial networks)^23^ proposed a 1D GAN to classify HSIs. However, this HSGAN uses features from only one layer in the GAN and does not mine enough information from HSIs.

In this paper, we propose an improved GAN method with multilayer convolutional features based on spectral angle distance (SAD) referred to as SADGAN. Considering the characteristics of the spectrum, our method uses an improved loss function in the GAN for hyperspectral image classification optimization. In addition, we use a multilayer convolutional neural network to classify the features from the GAN.

SAD is a spectral matching method that can be used to compare image spectra directly^32^. It is widely used in hyperspectral imagery unmixing^33^, hyperspectral image analysis^34^, and computer vision applications^35^. Since the SAD method can quickly calculate differences between the generated spectrum and real spectrum, we use SAD as the objective function to improve the convergence speed of the GAN. The generator model of the GAN can also generate samples closer to real data.

In the SADGAN, we designed a GAN with a 1D structure to extract spectral features. Then, the 1D GAN, with spectral angle distance as the loss function of $G$, is trained using the unlabeled samples. Once the entire model has been trained, the discriminative model $D$ will contain some multiscale filters to extract features^28^. Next, by inputting labeled samples into $D$, the $G$ model can obtain the features of the samples through convolution layers (filters). These features are flattened, concatenated and sent to a small CNN for training. Finally, we obtain a semisupervised learning model for HSI classification. The experimental results show that compared with state-of-the-art methods, the proposed semisupervised framework achieves competitive results.

The main contributions of this paper are as follows:

- (1)We used spectral angle distance as the objective function of the generator in the GAN. Compared with the common cross-entropy loss function, the spectral angle distance loss function can accelerate convergence of the GAN, make model training faster and generate data more similar to real data.
- (2)We create a new semisupervised classifier using the multilayer features in the discriminator of the GAN that can effectively classify HSIs by using a few labeled samples.

The rest of this paper is organized as follows. The “Proposed method” section presents the proposed semisupervised HSI classification method in detail. The “|Experiment” section reports the experimental results, the visualization of the intermediate results and the structural details of the GAN model on four benchmark HSI datasets. Conclusions and discussions are presented in the “Conclusion” section.

## Proposed method

### Generative adversarial networks

Since its proposal by Goodfellow et al.^25^, the GAN has become a popular deep learning method and has achieved great success with many visual generation tasks. Because of its ability to acquire features from both labeled and unlabeled samples, the GAN also represents a great breakthrough in semisupervised learning.

**Figure 1:**
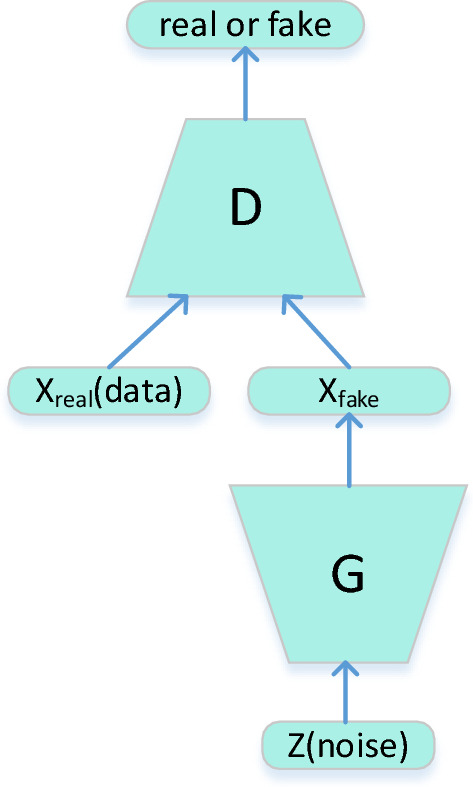
Architecture of the GAN.

The GAN is composed of two adversarial players, as shown in Fig. 1: a generative model $G$ that generates samples with the same distribution as that of the real samples, and a discriminative model $D$ that determines whether the samples generated by $G$ are real or fake. From a data point of view, the input of $G$ is the noise ${p}_{z}$, the output is the generated samples, and the distribution ${p}_{g}$ tries to imitate the distribution of the real data ${p}_{r}$. Moreover the input of $D$ is the fake samples generated by $G$ or the real sample, and the output of $D$ is the judgment of whether the data are real or fake^36^.

$G$ builds a mapping function from ${p}_{z}$ to a data space as $G(z;{\theta }_{g})$, which is a differentiable function with parameters ${\theta }_{g}$ that will learn the distribution ${p}_{r}$ over real data. A second function $D(x;{\theta }_{d})$ with parameters ${\theta }_{d}$ outputs a single scalar that discriminates whether a sample originated from the training data or $G$. $D(x)$ represents the probability that $x$ came from the real data rather than ${p}_{g}$. The training procedure involves solving the following minmax problem^25^:

1 $$\begin{array}{c}\,\underset{{\text{G}}}{{{\min}}}\underset{{\text{D}}}{{{\max}}}V\left(D,G\right)={{\text{E}}}_{{\text{x}} \sim {{\text{p}}}_{{\text{r}}}}\left[{{\text{logD}}}\left({{\text{x}}}\right)\right]+{E}_{z\sim {p}_{z}}\left[{{\text{log}}}\left(1-D\left(G\left(z\right)\right)\right)\right],\end{array}$$

where it is if $D$ and $G$ are playing the min–max game with value function $V\left(D,G\right)$, ${p}_{z}$ is a uniform distribution ${p}_{z}=\upmu \left(-{1,1}\right)$, and $E$ indicates expectation.

In practice, models $D$ and $G$ are trained alternatively in each training iteration. First, $D$ will be updated by ascending its stochastic gradient:

2 $$\begin{array}{c}{\nabla }_{{\uptheta }_{d}}\frac{1}{m}\sum_{i=1}^{m}\left[{\text{log}}D\left({x}^{\left(i\right)}\right)+{\text{log}}\left(\left\{1-D\left(G\left({z}^{\left(i\right)}\right)\right\}\right)\right)\right].\end{array}$$

After $D$ is trained several times, we can fix the parameters of the $D$ model and then update the parameters of the $G$ model by descending its stochastic gradient:

3 $$\begin{array}{c}{\nabla }_{{\uptheta }_{g}}\frac{1}{m}\sum_{i=1}^{m}{\text{log}}\left(\left\{1-D\left(G\left({z}^{\left(i\right)}\right)\right\}\right)\right).\end{array}$$

After several steps of training, $D$ is trained to discriminate samples from data, converging to

4 $$\begin{array}{c}{D}_{G}^{*}\left(x\right)=\frac{{p}_{r}\left(x\right)}{{p}_{r}\left(x\right)+{p}_{g}\left(x\right)}.\end{array}$$

$D$ and $G$ will reach a point at which both cannot be improved because ${p}_{g}={p}_{r}$ and $D$ is unable to differentiate between the two distributions.

Both $D$ and $G$ can be nonlinear mapping functions, such as multiplyer perceptrons^25^.

### Framework of the SADGAN

**Figure 2:**
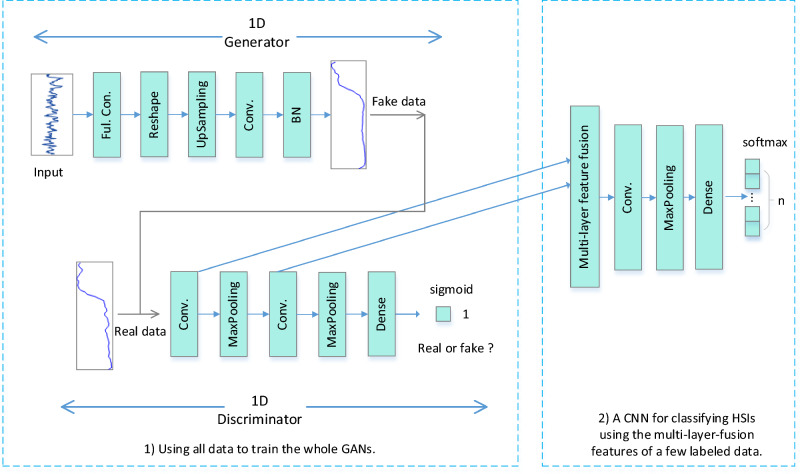
Framework of the proposed SADGAN.

The framework of the proposed SADGAN method is shown in Fig. 2 and consists of two parts: (1) a 1D GAN for spectral feature extraction and (2) a CNN for spectral classification. First, we use a custom 1D GAN for all the labeled and unlabeled data to obtain spectral features. Then, a small CNN will be trained by the features from a small number of labeled samples. The input of the CNN is the fusion of the multilayer features of the $D$ model in the GAN, and the output is the label of the sample. The individual steps in the method are outlined in the next discussions.

The HSGAN^23^ was proposed for the 1D hyperspectral GAN, which does not use all available features, and fine-tuning takes too much time because it does not take into account the characteristics of the spectra. To improve semisupervised classification, we proposed a semisupervised classification method based on a 1D GAN by adding multilayer features and spatial features.

As shown in Fig. 2, we designed a 1D GAN for hyperspectral semisupervised classification based on the structure of DCGAN^26^. The 1D generator $G$ takes the uniform noise distribution ${p}_{z}$ as the input to the fully connected (Ful. Con.) layer, whose output is reshaped into a 2D tensor. The upsampling layer is used to represent the inverse max pooling layer to reamplify the front layer to the needed size. The Conv. layer is a convolutional layer in the CNN that is used to extract the features. The batch normalization (BN) layer follows the convolution layer and stabilizes learning by normalizing the input of each unit^37^. The output of the last layer is the generated sample, which is provided as a “false” input to the $D$ model.

When the model is trained on all samples, $D$ can extract the features of all unlabeled and labeled samples. As shown in the lower left corner of Fig. 2, the 1D discriminator is also a CNN model that typically consists of several stacks with three parts: a convolution layer, a max pooling layer and a nonlinear mapping layer. The input of the $D$ model consists of two parts: one is a batch of real samples, and the other is a batch of “fake” samples generated by the $G$ model. The role of the $D$ model is to determine whether the input sample is real or fake. The convolutional (Conv. in Fig. 2) layer is a 1D convolution with a size of 5 $\times$ 1 or 3 $\times$ 1. All of the real and fake samples are 1D hyperspectral bands, which can be computed by the Conv. layer and output into the feature maps. The nonlinear mapping layer consists of a nonlinear activation function for each feature map to output the result that can activate the function. This layer is usually integrated with convolutional and max pooling layers, so we cannot draw it in Fig. 2. The max pooling layer can reduce the dimension of the feature map. In the $D$ model, the 1D input data are processed using a 1D max pooling of size 2 $\times$ 1 or 3 $\times$ 1.

When the model is trained on all the samples, $D$ will extract the features of all of the samples, and we can use these extracted features for spectral classification.

### Spectral angle distance loss function

The traditional GAN uses the binary loss function as the last layer of the $D$ model to train the GAN. For brevity, let $a$ = output and $b$ = target where $n$ is the dimension. The binary cross entropy loss is then

5 $$\begin{array}{c}loss\left({\varvec{a}},{\varvec{b}}\right)=-{\sum }_{i=1}^{n}\left[{a}_{i}{\text{log}}\left({b}_{i}\right)+\left(1-{a}_{i}\right){\text{log}}\left(1-{b}_{i}\right)\right].\end{array}$$

In the training process, through the loss obtained by this objective function, the parameters of the entire GAN can be continuously updated, and the $G$ model outputs the “real” data.

Equation (1) is the objective function of the traditional GAN, which Arjovsky et al.^38^ believe has the following two problems: (1) a well-trained discriminator model will lead to vanishing gradients and (2) the loss function is equivalent to an unreasonable distance metric. These are the root causes of why the traditional GAN is unstable during training and undergoes model collapse.

Considering the characteristics of the spectral curve, we introduce the spectral angle distance (SAD) as the loss function to train the $G$ model to improve the training efficiency of the 1D GAN. Correspondingly, the objective function^25^ of the GAN with SAD loss is:

6 $$\begin{array}{c}\underset{G}{{\text{min}}}\,\underset{D}{{\text{max}}}V\left(D,G\right)={E}_{x\sim {p}_{r}}\left[{\text{log}}D\left(x\right)\right]+{E}_{z\sim {p}_{z}}\left[{\text{log}}\left(1-D\left(G\left(z\right)\right)\right)\right]+{E}_{z\sim {p}_{z}}\left[-SAD\left(x,G\left(z\right)\right)\right].\end{array}$$

SAD is a method by which the similarity of a spectrum to a reference spectrum is determined by calculating the spectral angle. Each pixel in a hyperspectral image is treated as an $n$-dimensional vector, where $n$ is equal to the number of spectral bands^39^. In general, a smaller angle means that the spectrum is a closer match to the reference spectrum.

Given two spectra with $n$ bands, the angle $\uptheta$ between a target spectrum ${\varvec{a}}$ and a reference spectrum ${\varvec{b}}$ can be calculated by

7 $$\begin{array}{c}\theta ={\text{arccos}}\left(\frac{{{\varvec{a}}}^{\text{T}}{\varvec{b}}}{|{\varvec{a}}||{\varvec{b}}|}\right),\end{array}$$

where $| |$ is a norm function. According to Eq. (7), the SAD can be expressed by:

8 $$\begin{array}{c}SAD=\mathrm{cos\theta }=\frac{\sum_{i=1}^{n}{a}_{i}\cdot {b}_{i}}{\sqrt{\sum_{i=1}^{n}{a}_{i}^{2}}\sqrt{\sum_{i=1}^{n}{b}_{i}^{2}}},\end{array}$$

where ${a}_{i}$ and ${b}_{i}$ are the mean values of the spectra at the $i$ th band.

9 $$\begin{array}{c}Loss\_SAD=\frac{-\sum_{i=1}^{n}{a}_{i}\cdot {b}_{i}}{\sqrt{\sum_{i=1}^{n}{a}_{i}^{2}}\sqrt{\sum_{i=1}^{n}{b}_{i}^{2}}}\end{array}$$

The loss function of $G$ in the SADGAN will be replaced by Eq. (9), which is the opposite of Eq. (8). The smaller the value obtained by Eq. (9) is, the more similar the two spectra are. This equation will be used in the last layer of the $G$ model to calculate the loss of the spectrum generated by $G$ and the real spectrum.

We also need to calculate the derivative or gradient of Eq. (9) and pass it back to the previous layer during backpropagation^40^. The derivative of $SADLoss$ with respect to ${a}_{i}$ and ${b}_{i}$ can be calculated by Eq. (10) or (11):

10 $$\begin{array}{c}\frac{\partial Loss\_SAD}{\partial {a}_{i}}=\frac{-{b}_{i}}{\sqrt{\sum_{i=1}^{n}{a}_{i}^{2}}\sqrt{\sum_{i=1}^{n}{b}_{i}^{2}}}+\frac{{a}_{i}\sum_{i=1}^{n}{a}_{i}{b}_{i}}{{\left(\sqrt{\sum_{i=1}^{n}{a}_{i}^{2}}\right)}^{3}\sqrt{\sum_{i=1}^{n}{b}_{i}^{2}}},\end{array}$$

11 $$\begin{array}{c}\frac{\partial Loss\_SAD}{\partial {b}_{i}}=\frac{-{a}_{i}}{\sqrt{\sum_{i=1}^{n}{a}_{i}^{2}}\sqrt{\sum_{i=1}^{n}{b}_{i}^{2}}}+\frac{{b}_{i}\sum_{i=1}^{n}{a}_{i}{b}_{i}}{\sqrt{\sum_{i=1}^{n}{a}_{i}^{2}}{\left(\sqrt{\sum_{i=1}^{n}{b}_{i}^{2}}\right)}^{3}}.\end{array}$$

Equation (10) or (11) can be briefly written as Eq. (12) or (13):

12 $$\begin{array}{c}\frac{\partial Loss\_SAD}{\partial {a}_{i}}=\frac{-{b}_{i}}{|{\varvec{a}}||{\varvec{b}}|}+\frac{{a}_{i}\left({\varvec{a}}\cdot {\varvec{b}}\right)}{|{\varvec{a}}{|}^{3}|{\varvec{b}}|},\end{array}$$

13 $$\begin{array}{c}\frac{\partial Loss\_SAD}{\partial {b}_{i}}=\frac{-{a}_{i}}{|{\varvec{a}}||{\varvec{b}}|}+\frac{{b}_{i}\left({\varvec{a}}\cdot {\varvec{b}}\right)}{|{\varvec{a}}||{\varvec{b}}{|}^{3}}.\end{array}$$

From Eqs. (6) and (9), a new GAN loss function based on the SAD can be obtained:

14 $$\begin{array}{c}loss=\frac{1}{m}\sum_{i=1}^{m}{\text{log}}D\left({x}^{\left(i\right)}\right)+\frac{1}{m}\sum_{i=1}^{m}{\text{log}}\left(1-D\left(G\left({z}^{\left(i\right)}\right)\right)\right)-\frac{1}{m}\sum_{i=1}^{m}\frac{G{\left({z}^{\left(i\right)}\right)}^{T}\cdot {x}^{\left(i\right)}}{|G\left({z}^{\left(i\right)}\right)|\cdot |{x}^{\left(i\right)}|},\end{array}$$

where ${x}^{\left(i\right)}$ and ${z}^{\left(i\right)}$ are the real sample and noise sample during training, respectively, and $m$ is the training batch size. The loss function of the discriminator $D$ is:

15 $$\begin{array}{c}los{s}_{D}=\frac{1}{m}\sum_{i=1}^{m}{\text{log}}D\left({x}^{\left(i\right)}\right)+\frac{1}{m}\sum_{i=1}^{m}{\text{log}}\left(1-D\left(G\left({z}^{\left(i\right)}\right)\right)\right).\end{array}$$

The loss function of the generator $G$ is:

16 $$\begin{array}{c}los{s}_{G}=\frac{1}{m}\sum_{i=1}^{m}{\text{log}}\left(1-D\left(G\left({z}^{\left(i\right)}\right)\right)\right)-\frac{1}{m}\sum_{i=1}^{m}\frac{G{\left({z}^{\left(i\right)}\right)}^{T}\cdot {x}^{\left(i\right)}}{|G\left({z}^{\left(i\right)}\right)|\cdot |{x}^{\left(i\right)}|}.\end{array}$$

The discriminator can be updated by ascending its stochastic gradient^25^:

17 $$\begin{array}{c}{\nabla }_{{\uptheta }_{d}}\left(\frac{1}{m}\sum_{i=1}^{m}{\text{log}}D\left({x}^{\left(i\right)}\right)+\frac{1}{m}\sum_{i=1}^{m}{\text{log}}\left(1-D\left(G\left({z}^{\left(i\right)}\right)\right)\right)\right).\end{array}$$

The generator can be updated by descending its stochastic gradient:

18 $$\begin{array}{c}{\nabla }_{{\uptheta }_{g}}\frac{1}{m}\sum_{i=1}^{m}{\text{log}}\left(1-D\left(G\left({z}^{\left(i\right)}\right)\right)\right)-\frac{1}{m}\sum_{i=1}^{m}\frac{G{\left({z}^{\left(i\right)}\right)}^{T}\cdot {x}^{\left(i\right)}}{|G\left({z}^{\left(i\right)}\right)|\cdot |{x}^{\left(i\right)}|}.\end{array}$$

Using the SAD loss function, the whole process of training the GAN can be guided by the real samples in the generator process, the whole training process can converge faster, and the training is more stable.

### A small CNN for spectral classification with multilayer features

To classify HSIs in a semisupervised manner, HSGAN^23^ transformed the well-trained $D$ model into a classification network by replacing the top layer of $D$ with a softmax layer. However, this method uses only one-layer features. For better classification, we designed a small CNN that contains one convolutional layer with a size of $3 \times 1$ or $5 \times 1$, one max pooling layer with a size of $2 \times 1$, and a softmax layer to output the labels. The input of the CNN is a fusion of the multilayer features extracted from the $D$ model, and the output is the class of samples.

As shown in the center of Fig. 2, we trained the model on all HSI samples and then used the discriminator's convolutional features from the output of the convolutional layers. During the CNN training phase, a small number of labeled samples are fed into the discriminator, and then the convolutional features created by the $D$ model are flattened and concatenated to form a vector. This vector is input into the CNN with a softmax classifier on the top to output the labels.

The input of the CNN is the 1D hyperspectral feature vector of each pixel, which can be expressed as $x$. $x$ can be convoluted by $k$ 1D convolutional kernels $\left(n\times 1\right)$, and then we will obtain $k$ feature maps $y$:

19 $$\begin{array}{c}y=\sum_{i=1}^{k}{W}_{i}*{x}_{i}+b,\end{array}$$

where $y$ is the output feature map, $W$ is the weight in the $k$ th convolution, $*$ is a convolution operator, and ${\varvec{b}}$ is the bias.

Training the CNN requires two steps: forward propagation and back propagation. Each forward operation is followed by a backward operation. In the forward propagation phase, a batch of sample feature vectors are input to the CNN. The softmax layer outputs the probability of a sample belonging to a certain class^41^.

The difference between the label and the results of the forward process is calculated in the backpropagation process. Then, the weights and biases of the entire network are updated by using the gradient descent algorithm to minimize the loss function. In this paper, we use the least-squares loss function^42^:

20 $$\begin{array}{c}J\left(\uptheta \right)=\sum_{i=1}^{N}\left(\frac{1}{2}|Y\left({x}_{i},\uptheta \right)-\upgamma {|}^{2}\right)+\lambda \sum_{l}^{L}sum\left(|{\uptheta }^{\left(l\right)}{|}^{2}\right),\end{array}$$

where $Y$ denotes the result of the CNN, and $\upgamma$ denotes the target label.

After training with a small amount of labeled data, the CNN can be used to perform HSI classification.

### Algorithm overview: the training and classifying phase of the SADGAN

The SADGAN algorithm consists of three steps: 1D-GAN training, CNN classifier training, and classification. As shown in Algorithm 1, $G$ and $D$ are trained simultaneously using all of the unlabeled spectral curves. During training, $G$ generates fake spectral samples, and $D$ determines whether these samples are real. At the end of the training, we will obtain a trained $G$ that can generate data similar to the real data as well as a well-trained $D$ that can extract the features of all unlabeled samples. In the CNN training phase, the features are extracted from the outputs of some convolutional layers in the discriminator and then sent into the CNN. Later, the CNN is trained on a few labeled samples and is then used to classify the HSIs.

**Algorithm 1:**
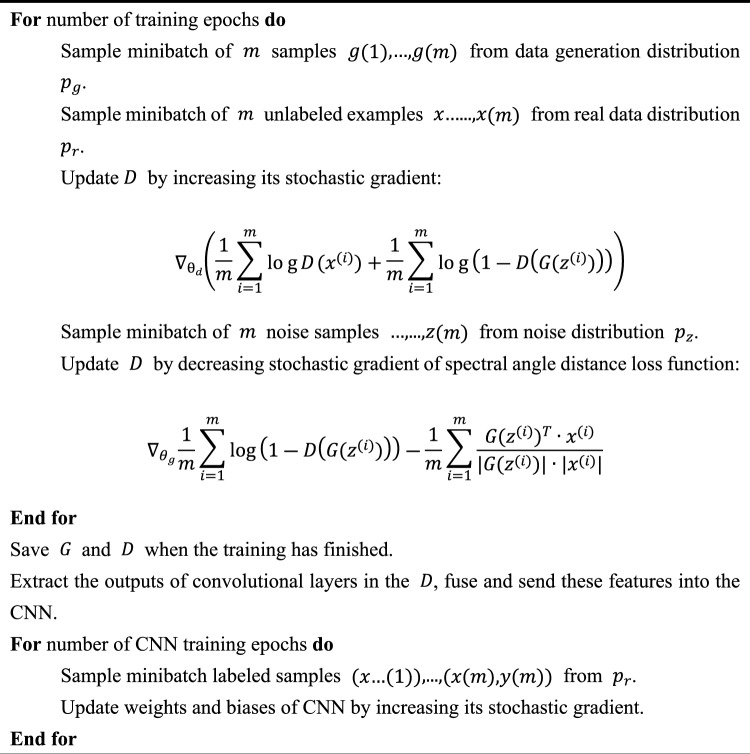
Training and classifying of the SADGAN.

## Experiment

The proposed method (SADGAN) was tested and compared with four hyperspectral datasets: the University of Pavia dataset, the Indian Pines dataset, the Salinas dataset related to agriculture, and the Tianshan dataset related to geological bodies.

**Figure 3:**
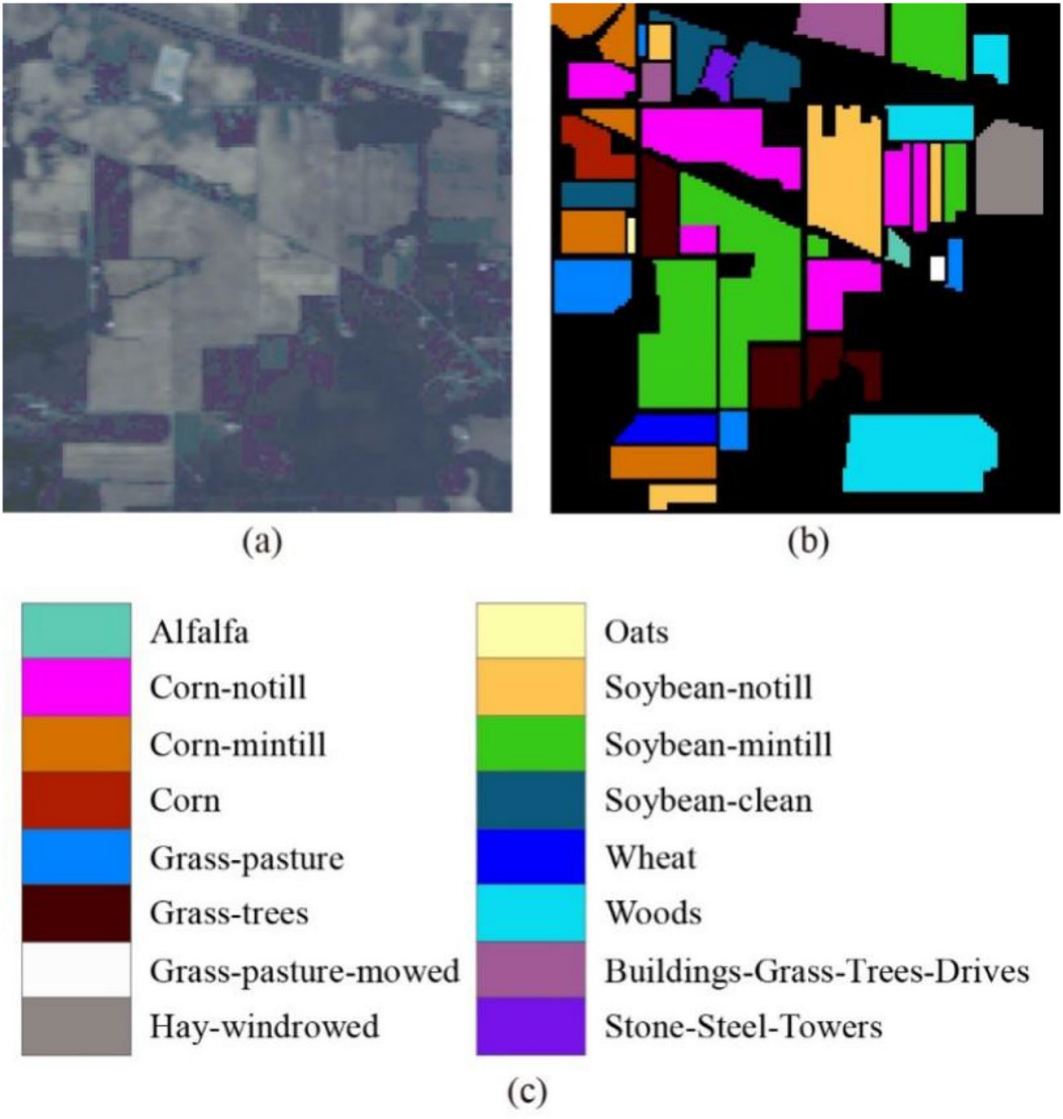
(**a**) Three-band color composite of the Indian Pines image. (**b**,**c**) Ground truth data.

The first hyperspectral dataset is Indian Pines, which was acquired by the airborne visible/infrared imaging spectrometer (AVIRIS) sensor over the Indian Pines region in northwestern Indiana in 1992. The image has a size of $145 \times 145$ pixels with 220 spectral bands covering the range from 400 to 2200 nm with fine spectral resolution. The spatial resolution is approximately 20 m. The water absorption and noisy bands were removed before the experiments, leaving 200 bands. A total of 10249 pixels from sixteen classes were used for our experiments. Figure 3 shows the color composite of the Indian Pines image and the corresponding ground truth data.

**Figure 4:**
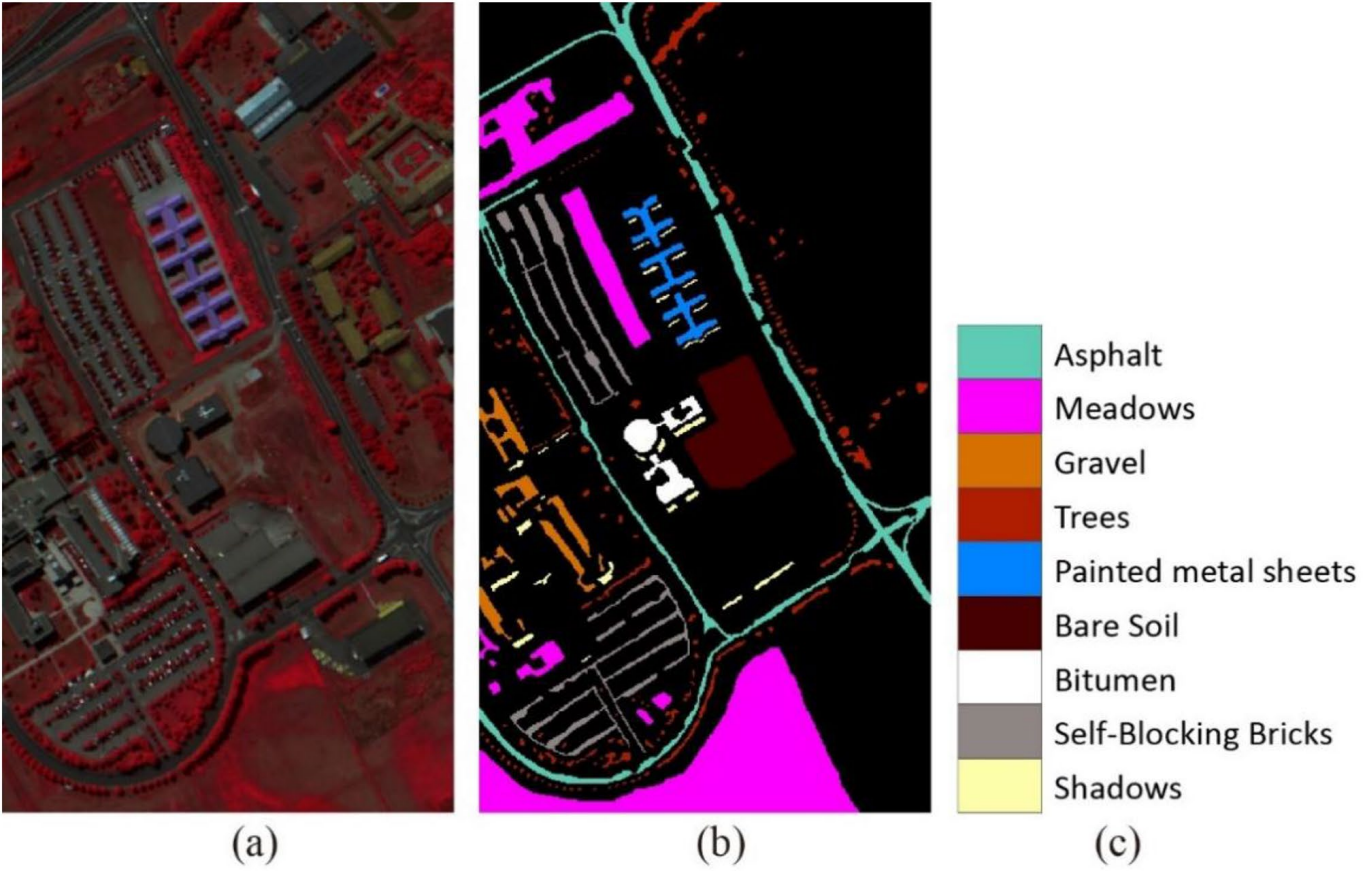
(**a**) Three-band color composite of the Pavia University image. (**b**) and (**c**) Ground truth data.

The second hyperspectral image was acquired by the airborne reflective optics system imaging spectrometer (ROSIS) sensor over the urban area of the University of Pavia, northern Italy, in 2002. The image size in pixels is 610 $\times$ 340. The image set has a high spatial resolution of 1.3 m and spectral coverage of 0.43 to 0.86 m. The number of bands in the acquired image is 103. There are a total of 42,776 samples in 9 categories. Figure 4 shows the color composite of the Pavia University image and the corresponding ground truth data.

**Figure 5:**
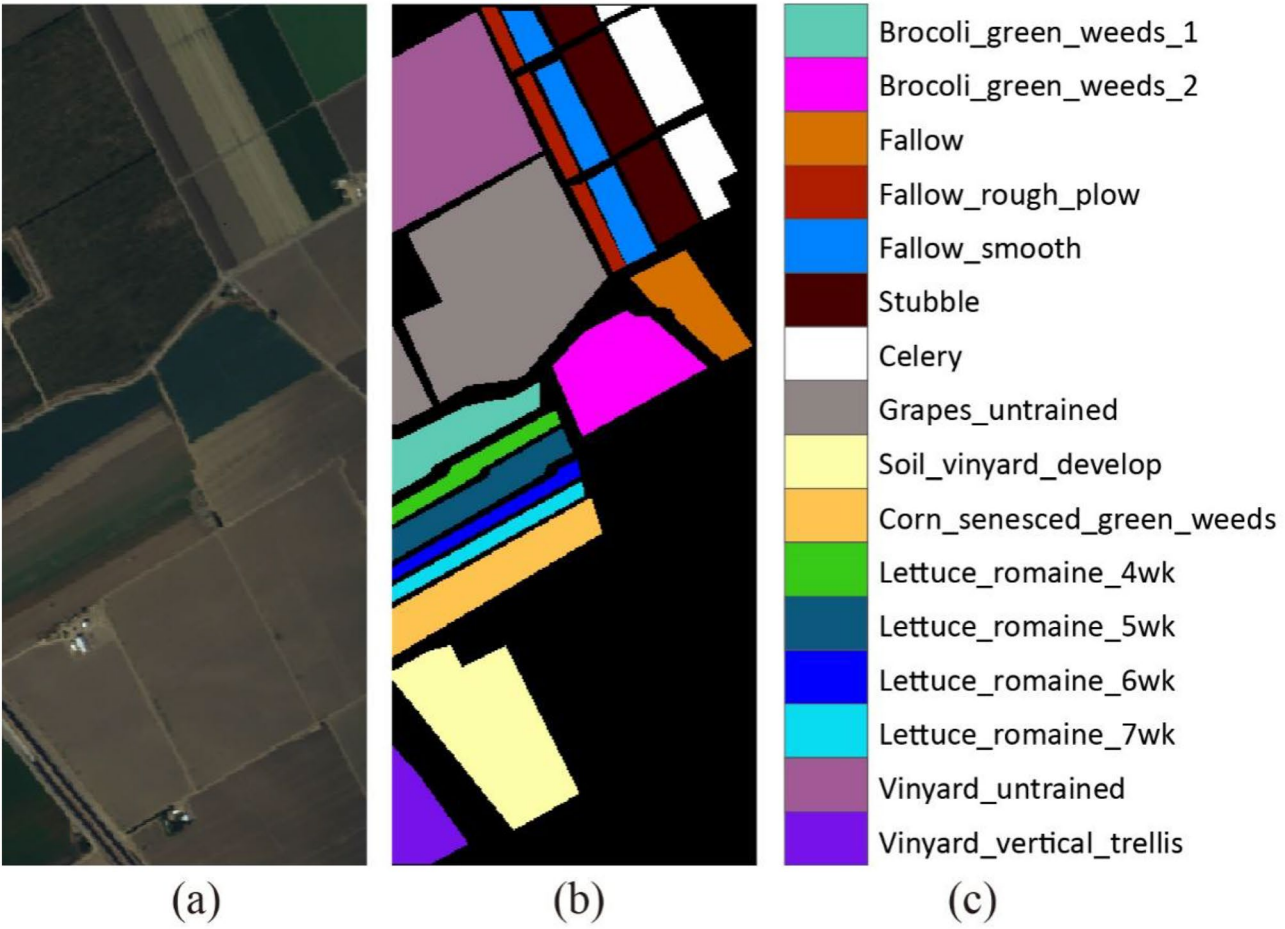
(**a**) Three-band color composite of the Salinas image. (**b**,**c**) Ground truth data.

The third set of hyperspectral data was collected by the AVIRIS sensor over the Salinas Valley in southern California, USA, in 1998. The image size is 512 $\times$ 217 pixels, the coverage is 400 to 2500 nm, and the spatial resolution is 3.7 m. After removing the noisy and water absorption bands, the number of bands in the acquired image is 204. There are a total of 54,129 samples in 16 classes. Figure 5 shows the color composite of the Salinas image and the corresponding ground truth data.

**Figure 6:**
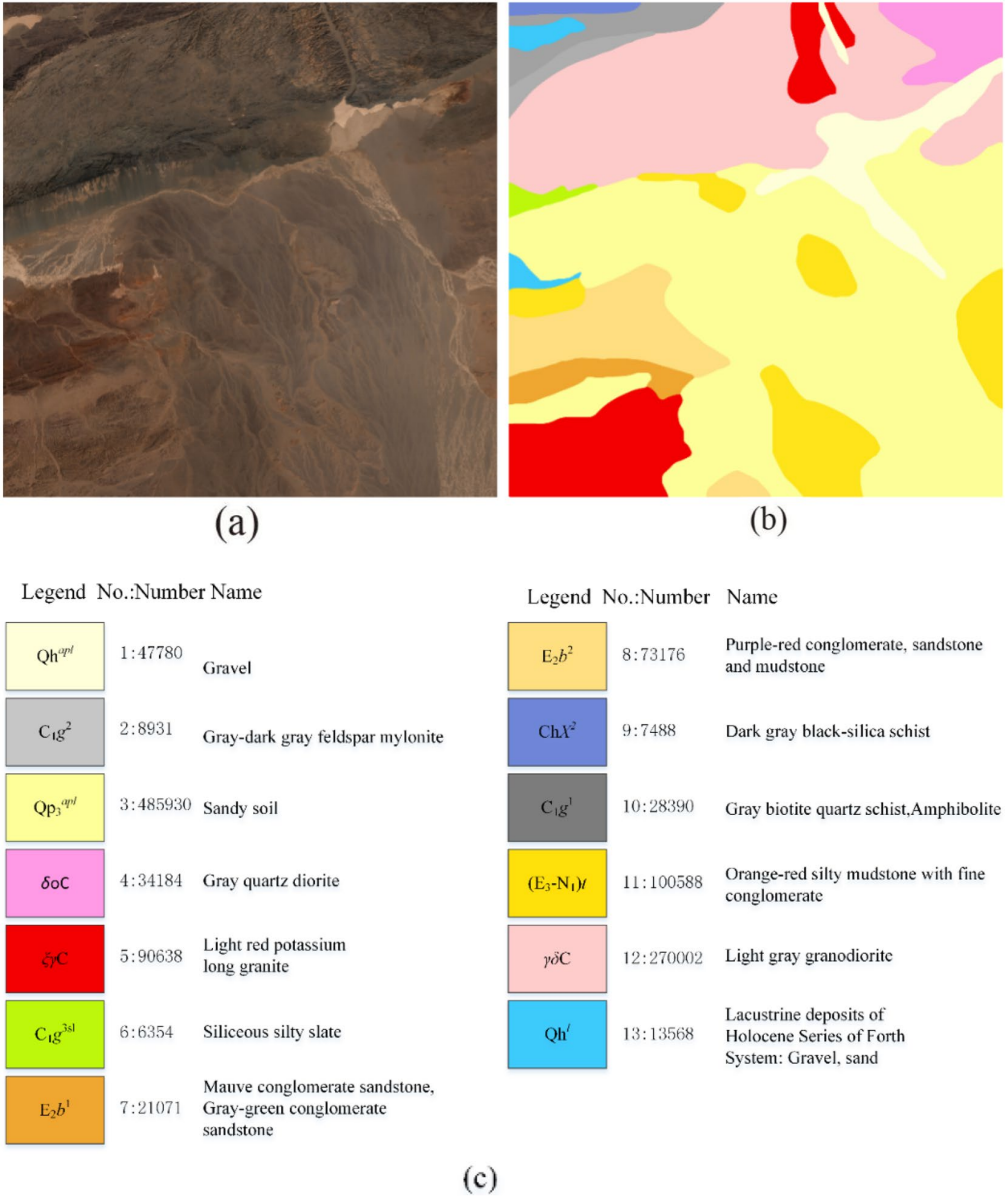
(**a**) Three-band color composite of the Tianshan image. (**b**,**c**) Ground truth and number of samples.

The fourth dataset is the airborne HyMap data acquired over Tianshan, China, in 2013. The spectral range of the HyMap imaging spectrometer is 0.40–2.48 µm, and the spectral bandwidth of the data is not fixed and generally between 15 and 18 nm, with an average bandwidth of approximately 16 nm and a spatial resolution of 9 m. The spectral response values of the features range from 1 to 10,000, and the experimental data have been atmospherically and geometrically corrected. A pixel area of 1090 × 1090 was selected as the study area, and after removing the water absorption and noise bands, 123 bands remained, of which 50 bands were selected as the final dataset using the BSCNN band selection method. Figure 6a shows the color composite map of the data in the study area. Figure 6b is the ground truth data map of the study area based on the existing local geological map, which is divided into 13 classes, and the number of samples in each class and the color legend are shown in Fig. 6c.

Based on the first three datasets, the performance of the proposed SADGAN method is compared with the performance of some state-of-the-art methods published recently. These methods include SVM^9^, BagRF^43^, RNN-LSTM^44^, 1DCNN^45^, DRNN^46^, GANs^30^, and HSGAN^23^. The SVM classifier used in the experiment comes from the Scikit-learn package. The kernel, $C$ and $\upgamma$ of the SVM are the default values in the package. BagRF is a classification technique for hyperspectral images based on random forest (RF) with the bagging method. LSTM (long short-term memory) has a RNN (recurrent neural network) architecture and was designed to better store and access information than the standard RNN. DRNN is a new RNN method based on deep learning that can effectively analyze hyperspectral pixels as sequential data and then determine the information categories via network reasoning. The structure of the 1DCNN method is the same as that of $D$ in the SADGAN, and its top layer will be replaced with the softmax layer with $n$ output classes. The GAN method was proposed for HSI classification^30^. HSGAN is a HSI classification method based on the GAN proposed in Ref.^30^, and the structures of the $D$ and $G$ models in HSGAN are consistent with the SADGAN method on the corresponding dataset.

The experiments were performed on an Intel Core i5-4590 3.3-GHz CPU, 20-GB random access memory, and a Titan X GPU. The proposed SADGAN method is conducted by the TensorFlow framework and the Keras library, and each result is the average of the classifier after ten runs. To quantitatively evaluate the experimental results, we compared these methods with three standard indicators, i.e., overall accuracy (OA), average accuracy (AA) and kappa coefficient ($\upkappa$). We used only unlabeled samples to train our GAN model. For comparison with more current state-of-the-art methods, each dataset uses a different number of training and testing datasets. In the experiments, a portion of the training samples are selected randomly as labeled samples, and the rest are used as unlabeled samples. The details and comparison methods are described in detail in the following sections.

### Experiments with the Indian Pines dataset

Table 1 shows the architecture of the SADGAN designed for the Indian Pines dataset. $G$ refers to the generator, $D$ refers to the discriminator and $C$ indicates the CNN. FC refers to the fully connected layer. BN denotes whether batch normalization^47^ was used. $G$ consists of layers 1 through 9, and $D$ consists of layers 10 through 18. Models $D$ and $G$ are trained simultaneously. The number of training epochs of the GAN is 100, and the batch size is 128. The optimizer is Adam, a first-order gradient-based optimizer of stochastic objective functions with a learning ratio of 0.001. All weights were initialized from a zero-centered normal distribution. Following Algorithm 1, we trained models $G$ and $D$ simultaneously. In the CNN training phase, we send $m$ labeled samples to the $D$ model, and we extract the output features of layers 11, 12, 14 and 15, whose shapes are (m, 32, 1, 198), (m, 32, 1, 197), (m, 32, 1, 97) and (m, 32, 1, 95), respectively. These features are concatenated into a new array with shape (m, 32, 1, 568) and used to train the CNN classifier. The CNN model with the highest classification accuracy will be saved. When the training of the CNN is complete, we will obtain a well-trained classifier for the Indian Pines dataset.

**Table 1:** Architecture of the SADGAN for the Indian Pines dataset.

	No	Layer	Kernel	Feature map	BN	Activation function
$G$	1	$G$-input	1 × 100	0	No	No
	2	FC	1 × 1024	1	No	tanh
	3	FC	1 × 50 × 128	1	Yes	tanh
	4	Reshape	1 × 50	128	No	No
	5	Upsampling	1 × 2	0	Yes	No
	6	Conv	1 × 5	64	No	tanh
	7	Upsampling	1 × 2	0	No	No
	8	Conv	1 × 5	32	No	tanh
	9	$G$-output	1 × 200	0	No	No
$D$	10	$D$-input	1 × 200	0	No	No
	11	Conv	1 × 3	32	No	Relu
	12	Conv	1 × 3	32	No	Relu
	13	Max pooling	1 × 2	1	No	No
	14	Conv	1 × 3	32	No	Relu
	15	Conv	1 × 3	32	No	Relu
	16	Max pooling	1 × 2	1	No	No
	17	FC	1 × 1024	1	No	No
	18	$D$-output	1 × 1	0	No	Sigmoid
$C$	19	$C$-input	32 × 586	0	No	Relu
	20	Conv	1 × 3	32	No	Relu
	21	Max pooling	1 × 2	1	No	No
	22	FC	1 × 1024	1	No	Relu
	23	$C$-output	1 × 16	0	No	Softmax

**Figure 7:**
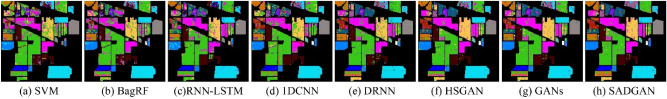
Classification results obtained by different methods for the Indian Pines scene.

In the CNN training and classification phase, we choose 10% of the labeled samples from each class. The experimental results shown in Table 2 demonstrate the superior performance of the proposed method (SADGAN). Compared to other methods, deep learning-based methods (1DCNN, DRNN, HSGAN, GANs and SADGAN) achieve higher classification accuracy. It is worth noting that the OAs of the 7th and 9th classes in the table based on the traditional methods SVM, BagRF, and RNN-LSTM have very low values, while other methods based on deep learning show a greatly improved distinction between these two classes. In these deep learning-based methods, the semisupervised methods (i.e., GANs, HSGAN and SADGAN) that consider the unlabeled training samples can generate samples closer to the actual spectral curve and perform better on classification tasks than other methods (1DCNN and DRNN) that use only the limited number of labeled training samples. The SADGAN method can use more convolution features extracted from the $D$ model, and it can achieve higher classification accuracy than the HSGAN method that uses only one layer of features. Figure 7 shows the classification results obtained by different methods for the Indian Pines scene.

**Table 2:** Comparison of the classification accuracies (%)of various methods for the Indian Pines dataset using 10% labeled training samples per class.

Class	SVM	BagRF	RNN-LSTM	1DCNN	DRNN	HSGAN	GANs	SADGAN
1	12.09 ± 10.54	17.62 ± 5.85	6.34 ± 2.72	**77.32** ± 12.44	49.27 ± 15.84	52.44 ± 4.91	68.05 ± 7.59	77.07 ± 5.69
2	55.66 ± 4.58	63.60 ± 4.51	69.29 ± 6.33	66.99 ± 8.61	83.73 ± 0.78	82.02 ± 0.38	79.26 ± 0.69	**86.58** ± 1.61
3	42.22 ± 5.39	51.49 ± 4.14	50.32 ± 2.24	60.88 ± 2.05	67.62 ± 7.51	75.93 ± 5.41	74.19 ± 1.14	**78.62** ± 0.45
4	18.40 ± 7.68	28.35 ± 4.55	22.49 ± 3.26	63.05 ± 10.90	52.77 ± 2.87	66.90 ± 5.16	60.47 ± 13.15	**84.32** ± 1.72
5	77.12 ± 2.98	77.43 ± 4.05	60.85 ± 9.03	89.22 ± 2.31	82.14 ± 1.07	83.78 ± 0.84	82.47 ± 0.88	**89.54** ± 2.02
6	95.93 ± 2.24	94.49 ± 3.57	94.82 ± 1.74	**97.75** ± 0.68	97.95 ± 0.37	95.04 ± 0.44	96.04 ± 1.11	97.56 ± 0.32
7	3.08 ± 8.03	14.80 ± 12.53	0.00 ± 0.00	87.20 ± 2.40	76.00 ± 5.93	88.80 ± 3.49	74.00 ± 2.00	**90.00** ± 2.00
8	98.24 ± 1.92	96.51 ± 3.01	97.84 ± 0.21	98.79 ± 0.34	97.40 ± 0.89	96.60 ± 0.19	96.67 ± 0.83	**99.12** ± 0.20
9	0.000.00	10.56 ± 8.03	0.00 ± 0.00	24.44 ± 5.09	25.56 ± 7.54	50.00 ± 8.24	41.11 ± 9.36	**52.22** ± 6.19
10	54.59 ± 7.28	64.69 ± 4.56	69.55 ± 7.09	73.42 ± 11.25	79.78 ± 0.85	70.88 ± 1.03	79.63 ± 3.52	**82.55** ± 0.82
11	85.95 ± 2.04	85.25 ± 1.87	87.94 ± 1.15	78.98 ± 6.90	82.58 ± 1.11	87.79 ± 2.58	**89.84** ± 0.76	89.46 ± 0.78
12	26.38 ± 3.07	43.49 ± 2.69	61.16 ± 1.65	68.22 ± 4.09	86.47 ± 3.86	79.96 ± 7.41	83.66 ± 3.26	**87.90** ± 0.47
13	95.46 ± 1.54	92.71 ± 2.48	92.72 ± 2.22	96.63 ± 0.80	97.83 ± 0.81	**99.24** ± 0.27	99.02 ± 0.47	98.80 ± 0.33
14	96.82 ± 1.13	95.04 ± 1.39	94.45 ± 2.05	**97.21** ± 0.17	95.73 ± 0.18	94.94 ± 0.76	97.16 ± 1.34	95.49 ± 1.20
15	24.67 ± 3.82	35.75 ± 3.67	9.16 ± 2.67	56.02 ± 2.99	40.75 ± 2.87	55.65 ± 7.56	58.56 ± 1.99	**73.98** ± 2.16
16	82.39 ± 3.91	78.94 ± 3.18	81.57 ± 1.53	**96.27** ± 0.84	77.95 ± 3.45	81.81 ± 3.02	83.86 ± 1.54	90.36 ± 1.52
OA	69.55 ± 1.02	73.43 ± 0.68	74.27 ± 0.34	78.87 ± 0.76	82.60 ± 0.66	83.84 ± 0.91	85.13 ± 0.59	**88.58** ± 0.12
AA	54.31 ± 1.28	59.42 ± 1.18	56.16 ± 0.69	77.02 ± 1.54	74.59 ± 0.46	78.86 ± 1.38	79.00 ± 1.13	**85.85** ± 0.77
$\kappa$	64.49 ± 1.23	69.29 ± 0.77	70.22 ± 0.41	75.80 ± 1.02	80.07 ± 0.73	81.46 ± 1.02	82.93 ± 0.69	**86.95** ± 0.14

Bold values indicate the best results.

### Experiments with the Pavia university dataset

Table 3 shows the architecture of our method for the Pavia University dataset. The number of training epochs of the GAN is 200, and the rest of the setup and workflow are the same as those for the Indian Pines dataset. After $G$ and $D$ are trained, we send $m$ labeled samples to the $D$ model to extract the output features of layers 11, 12, 14 and 15, whose shapes are (m, 32, 1, 101), (m, 32, 1, 99), (m, 32, 1, 48) and (m, 32, 1, 46), respectively. These features are concatenated into a new array with shape ($m$, 32, 1, 294) and used to train the CNN classifier $C.$ The number of CNN training epochs of $C$ is 5000, and the optimizer is stochastic gradient descent (SGD) with a learning rate of 0.0001. The CNN model with the highest accuracy is saved as the final classifier.

**Table 3:** Architecture of the SADGAN for the Pavia university dataset.

	No	Layer	Kernel	Feature map	BN?	Activation function
$G$	1	$G$-input	1 × 100	0	No	No
	2	FC	1 × 1024	1	No	tanh
	3	FC	1 × 26 × 64	1	Yes	tanh
	4	Reshape	1 × 26	64	No	No
	5	Upsampling	1 × 2	0	Yes	No
	6	Conv	1 × 5	32	No	tanh
	7	Upsampling	1 × 2	0	No	No
	8	Conv	1 × 5	32	No	tanh
	9	$G$-output	1 × 103	0	No	No
$D$	10	$D$-input	1 × 103	0	No	No
	11	Conv	1 × 3	32	No	Relu
	12	Conv	1 × 3	32	No	Relu
	13	Max pooling	1 × 2	1	No	No
	14	Conv	1 × 3	32	No	Relu
	15	Conv	1 × 3	32	No	Relu
	16	Max pooling	1 × 2	1	No	No
	17	FC	1 × 1024	1	No	No
	18	$D$-output	1 × 1	0	No	Sigmoid
$C$	19	$C$-input	32 × 294	0	No	Relu
	20	Conv	1 × 3	32	No	Relu
	21	Max pooling	1 × 2	1	No	No
	22	FC	1 × 1024	1	No	Relu
	23	$C$-output	1 × 9	0	No	Softmax

**Figure 8:**
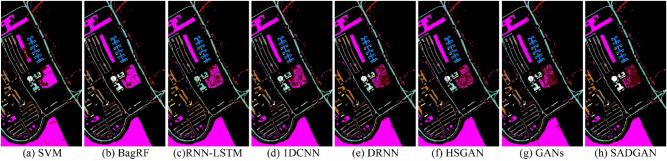
Classification results obtained by different methods for the Pavia University scene.

In the CNN training and classification phase, we choose 1% of the labeled samples from each class to train the CNN. Table 4 reports the overall accuracy (OA), average accuracy (AA), and $\upkappa$ statistic with their standard deviations after ten Monte Carlo runs. The experimental results demonstrate the superior performance of the proposed method (SADGAN). From the abovementioned results, the methods based on deep learning (i.e. 1DCNN, DRNN, HSGAN, GANs and SADGAN) achieve higher classification accuracy than the other methods. In the deep learning-based methods, the semisupervised methods (i.e. HSGAN, GAN and SADGAN) that consider the unlabeled training samples can perform better on classification tasks than 1DCNN, which uses only a limited number of labeled training samples. By using more features extracted from GAN, the proposed method SADGAN can achieve higher classification accuracy than HSGAN, which utilizes only one feature layer of the GAN. Figure 8 shows the classification results obtained by different methods for the Pavia University scene.

**Table 4:** Comparison of the classification accuracies (%) of various methods for the Pavia University dataset using 1% of the labeled training samples per class.

Class	SVM	BagRF	RNN-LSTM	1DCNN	DRNN	HSGAN	GANs	SADGAN
1	84.23 ± 5.85	**83.91** ± 3.95	82.98 ± 1.03	80.67 ± 0.64	81.58 ± 1.26	83.40 ± 1.55	76.51 ± 0.60	81.95 ± 2.20
2	95.54 ± 2.08	95.93 ± 1.69	97.56 ± 1.18	97.29 ± 1.70	97.73 ± 0.44	98.12 ± 0.98	**99.43** ± 0.09	99.13 ± 0.12
3	28.25 ± 10.99	34.32 ± 7.83	53.07 ± 10.69	49.60 ± 3.57	**71.91** ± 13.79	67.07 ± 4.97	51.53 ± 15.23	70.77 ± 1.18
4	75.71 ± 8.02	76.76 ± 6.58	71.47 ± 1.75	70.79 ± 8.89	72.47 ± 2.81	73.50 ± 4.47	62.21 ± 7.81	**79.56** ± 0.30
5	96.35 ± 4.59	94.32 ± 7.62	97.16 ± 0.08	96.82 ± 0.79	97.75 ± 0.74	97.57 ± 0.05	97.82 ± 0.23	**98.12** ± 0.08
6	27.00 ± 5.95	37.50 ± 3.74	50.07 ± 5.02	56.34 ± 1.19	71.34 ± 0.64	71.46 ± 0.30	79.82 ± 1.14	**83.43** ± 1.23
7	25.11 ± 26.65	62.20 ± 11.33	7.26 ± 6.81	51.92 ± 8.38	49.32 ± 13.43	53.19 ± 13.12	**91.10** ± 0.05	90.65 ± 0.28
8	80.99 ± 9.07	82.59 ± 4.77	80.12 ± 2.69	84.95 ± 1.55	79.48 ± 7.08	87.63 ± 1.31	**95.24** ± 2.65	94.52 ± 0.43
9	**99.69** ± 0.15	99.20 ± 0.27	97.78 ± 0.22	98.57 ± 1.43	96.93 ± 1.39	97.25 ± 0.75	98.79 ± 0.27	99.06 ± 0.11
OA	77.68 ± 1.09	80.63 ± 1.05	81.35 ± 0.88	83.20 ± 0.20	85.96 ± 0.37	87.08 ± 0.39	87.87 ± 0.04	**91.13** ± 0.39
AA	68.10 ± 3.26	74.08 ± 2.51	70.83 ± 0.68	76.33 ± 1.03	79.84 ± 2.36	81.02 ± 1.66	83.60 ± 0.59	**88.58** ± 0.24
κ	69.24 ± 1.54	73.47 ± 1.48	74.40 ± 1.20	77.10 ± 0.11	80.98 ± 0.57	82.50 ± 0.60	83.61 ± 0.07	**88.11** ± 0.52

Bold values indicate the best results.

### Experiments with the Salinas dataset

Table 5 shows the architecture of the SADGAN designed for the Salinas datasets. We trained the model $G$ and $D$ simultaneously following Algorithm 1. The number of training epochs of the GAN is 100, and the remaining architecture is the same as that for the Pavia University and Indian Pines datasets. In the CNN training phase, we send $m$ labeled samples to the $D$ model and extract the output features of layers 11, 12, 14 and 15, whose shapes are (m, 32, 1, 202), (m, 32, 1, 200), (m, 32, 1, 99) and (m, 32, 1, 97), respectively. These features are concatenated into a new array with shape (m, 32, 1, 598) and used to train the CNN classifier. The CNN model with the highest classification accuracy will be saved.

**Table 5:** Architecture of SADGAN for the salinas dataset.

	No	Layer	Kernel	Feature map	BN?	Activation function
$G$	1	$G$-input	1 × 100	0	No	No
	2	FC	1 × 1024	1	No	tanh
	3	FC	1 × 51 × 128	1	Yes	tanh
	4	Reshape	1 × 51	128	No	No
	5	Upsampling	1 × 2	0	Yes	No
	6	Conv	1 × 5	64	No	tanh
	7	Upsampling	1 × 2	0	No	No
	8	Conv	1 × 5	32	No	tanh
	9	$G$-output	1 × 204	0	No	No
$D$	10	$D$-input	1 × 204	0	No	No
	11	Conv	1 × 3	32	No	relu
	12	Conv	1 × 3	32	No	relu
	13	Max pooling	1 × 2	1	No	No
	14	Conv	1 × 3	32	No	Relu
	15	Conv	1 × 3	32	No	Relu
	16	Max pooling	1 × 2	1	No	No
	17	FC	1 × 1024	1	No	No
	18	$D$-output	1 × 1	0	No	Sigmoid
$C$	19	$C$-input	32 × 598	0	No	Relu
	20	Conv	1 × 3	32	No	Relu
	21	Max pooling	1 × 2	1	No	No
	22	FC	1 × 1024	1	No	Relu
	23	$C$-output	1 × 16	0	No	Softmax

**Figure 9:**
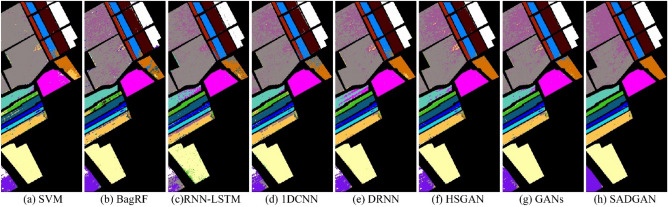
Classification results obtained by different methods for the salinas scene.

In the CNN training and classification phase, we choose 1% of the labeled samples from each class. Table 6 shows that the proposed method (SADGAN) performs much better than the other methods. Figure 9 shows the classification results obtained by different methods for the Salinas Valley scene.

**Table 6:** Comparison of the classification accuracies (%) of various methods for the Salinas dataset using 1% of the labeled training samples per class.

Class	SVM	BagRF	RNN-LSTM	1DCNN	DRNN	HSGAN	GANs	SADGAN
1	97.75 ± 1.02	97.18 ± 1.23	8.17 ± 7.38	90.81 ± 6.30	80.90 ± 14.32	**99.15** ± 0.05	96.02 ± 2.35	99.12 ± 0.72
2	97.67 ± 1.79	99.76 ± 0.14	98.81 ± 0.48	99.39 ± 0.39	99.62 ± 0.22	99.87 ± 0.03	99.67 ± 0.05	**99.99** ± 0.01
3	59.28 ± 14.03	81.56 ± 10.15	88.63 ± 9.08	88.05 ± 7.83	96.21 ± 1.64	91.59 ± 4.63	94.53 ± 2.11	**99.80** ± 0.00
4	98.30 ± 1.06	94.16 ± 2.58	93.21 ± 1.66	96.42 ± 1.64	98.46 ± 0.05	99.25 ± 0.10	98.53 ± 0.40	9**9.67** ± 0.04
5	96.20 ± 2.96	94.29 ± 3.86	**97.89** ± 0.25	94.29 ± 2.54	92.69 ± 0.71	93.52 ± 1.16	97.90 ± 0.06	97.86 ± 0.47
6	98.59 ± 0.85	96.58 ± 1.43	95.38 ± 0.26	97.61 ± 2.13	99.67 ± 0.05	99.57 ± 0.05	99.46 ± 0.10	**99.95** ± 0.03
7	99.28 ± 0.45	98.28 ± 0.79	99.30 ± 0.05	99.08 ± 0.15	98.95 ± 0.14	99.60 ± 0.10	99.79 ± 0.02	**99.92** ± 0.00
8	85.82 ± 10.39	80.44 ± 4.10	75.28 ± 3.47	81.66 ± 5.68	**90.61** ± 0.14	85.82 ± 1.29	85.74 ± 0.68	86.22 ± 3.64
9	98.65 ± 0.84	97.93 ± 0.90	97.97 ± 0.52	98.81 ± 0.14	98.79 ± 0.10	99.95 ± 0.03	99.81 ± 0.14	**99.96** ± 0.01
10	71.13 ± 9.58	75.88 ± 5.47	63.61 ± 4.04	77.52 ± 3.88	88.35 ± 0.37	88.08 ± 1.89	88.31 ± 0.73	**95.50** ± 1.10
11	78.77 ± 6.93	76.74 ± 6.86	88.53 ± 0.93	80.84 ± 8.64	86.78 ± 0.12	93.23 ± 0.29	86.89 ± 0.63	**96.58** ± 1.19
12	92.21 ± 7.93	92.31 ± 8.21	78.45 ± 11.26	98.27 ± 0.29	99.42 ± 0.13	99.84 ± 0.05	99.43 ± 0.11	**99.90** ± 0.05
13	**98.18** ± 1.30	96.85 ± 2.31	93.06 ± 0.92	93.13 ± 1.44	95.55 ± 0.13	96.98 ± 1.15	94.87 ± 3.48	97.69 ± 0.65
14	86.62 ± 4.52	89.97 ± 3.59	89.62 ± 3.37	94.36 ± 0.16	95.21 ± 0.25	93.00 ± 1.31	93.14 ± 1.08	**97.65** ± 0.07
15	27.50 ± 19.95	47.21 ± 6.57	62.12 ± 0.54	52.42 ± 7.91	42.13 ± 0.72	56.76 ± 1.61	63.10 ± 3.01	**73.40** ± 6.52
16	72.48 ± 12.25	85.30 ± 9.35	61.11 ± 3.70	73.32 ± 3.43	75.53 ± 3.68	93.74 ± 0.22	88.71 ± 2.46	**98.89** ± 0.33
OA	81.39 ± 1.35	84.08 ± 0.98	80.15 ± 1.65	85.31 ± 0.51	86.79 ± 0.38	89.23 ± 0.16	89.90 ± 0.19	**92.92** ± 0.29
AA	84.90 ± 1.93	87.78 ± 1.09	80.70 ± 1.74	88.50 ± 1.38	89.93 ± 0.76	93.12 ± 0.34	92.87 ± 0.09	**96.38** ± 0.47
κ	79.11 ± 1.57	82.21 ± 1.09	77.80 ± 1.86	83.56 ± 0.61	85.21 ± 0.43	87.97 ± 0.19	88.73 ± 0.21	**92.12** ± 0.33

Bold values indicate the best results.

### Experiments with the Tianshan dataset

The Tianshan dataset differs from the above three hyperspectral dataset related to agriculture and reflects the classification characteristics of hyperspectral remote sensing in geological bodies. To show the performance of the algorithms proposed in this paper in real hyperspectral image geological body classification applications, we select a variety of algorithms to classify geological bodies in hyperspectral images. These algorithms include SVM^9^, CNN^45^, HSGAN^23^, and SADGAN for spectral classification only, as well as SVM-EMP^48^, GIF-CNN^49^, SSGAT^50^, and GIF-SADGAN for spectral-spatial classification. The GIFs in GIF-SADGAN and GIF-CNN are consistent with each other, from paper^49^, denoting multiscale bootstrap map filtering.

Table 7 shows the comparison results of the classification performances of various methods with 10% of the training data. For the first four spectral classification methods, from the classification results, we can see that the SADGAN method proposed in this paper can achieve an overall classification accuracy of 86.58% in the hyperspectral images of geological bodies in the Tianshan dataset, which is 9.99 percentage points higher than the traditional SVM method, and the SADGAN method has a good classification performance. Among the deep learning methods CNN, HSGAN, and SADGAN, the semisupervised learning methods HSGAN and SADGAN, which can effectively utilize both labeled and unlabeled samples, perform better than the CNN. SADGAN utilizes multilayer convolutional features and performs slightly better than HSGAN. For spatial-spectral joint classification, although the nondeep learning traditional classification method SVM-EMP can utilize spatial features, its overall accuracy still lags behind that of other deep learning-based spectral-spatial classification methods and is even slightly lower than that of the spectral-only classification method SADGAN. GIF-SADGAN can perform better than GIF-CNN and SSGAT due to the excellent spectral classification performance of SADGAN.

**Table 7:** Comparison of the classification accuracies (%) of various methods for the Tianshan dataset using 10% labeled training samples per class.

Class	SVM	CNN	HSGAN	SADGAN	EMP-SVM	GIF-CNN	SSGAT	GIF-SADGAN
1	16.98	71.23	70.58	59.31	41.72	75.16	82.92	**89.44**
2	0.39	39.13	26.21	27.49	44.07	79.26	**83.47**	83.36
3	93.29	93.67	92.28	90.13	93.2	96.35	**96.68**	94.13
4	57.95	85.16	77.62	89.1	80.94	91.93	93.44	**93.53**
5	75.38	84.04	79.73	81.23	85.35	89.94	90.33	**92.40**
6	0.24	52.11	45.07	57.4	48.79	**88.90**	86.59	81.71
7	7.56	62.93	44.77	50.8	48.76	75.07	82.18	**84.66**
8	69.33	82.6	82.5	67.68	80.09	85.84	86.77	**93.70**
9	6.17	68.83	59.33	61.5	62.55	84.11	**87.18**	82.28
10	60.23	72.33	71.72	67.78	69.54	81.55	75.78	**82.28**
11	39.67	74.96	73.53	72.83	78.3	87.05	89.16	**95.06**
12	92.03	93.11	92.71	88.27	94.75	94.42	94.79	**96.43**
13	15.68	61.56	39.28	42.84	55.47	71.26	**87.73**	76.07
OA	76.59	82.31	85.04	86.58	85.88	91.74	92.80	**93.49**
AA	41.15	65.87	65.8	70.91	67.97	84.68	87.46	**88.08**
κ	67.46	77.53	80.06	82.08	81.04	89.06	90.49	**91.47**

Bold values indicate the best results.

**Figure 10:**
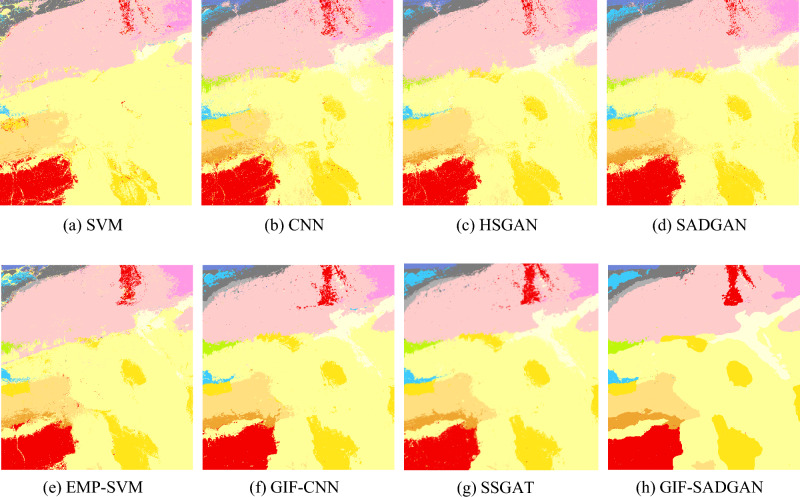
Classification results obtained by different methods for the Tianshan dataset.

Figure 10 shows maps of the comparison of the classification results from these experiments on the 50-band Tianshan dataset. Benefiting from the GAN's generator-generated (augmented) samples and multilayer convolutional features, SADGAN's classification result maps are more detailed. However, many cluttered spots exist in homogeneous regions because the spatial features are not utilized. From the classification results of the GIF-CNN, SSGAT, and GIF-SADGAN methods, it can be seen that because of the spatial feature extraction method, compared with the SADGAN, most of the clutter in the homogeneous region has been correctly categorized, which also demonstrates that the spectral-based method proposed in this paper can be combined with a spatial feature extraction method to achieve a better classification result. However, it should also be noted that the handmade real ground data will also have errors; although the accuracy of spectral classification, such as that of the SADGAN, is usually lower than that of spatial-spectral joint classification, it will also reflect the features of the ground features in a more detailed way. In contrast, the spatial-spectral joint classification algorithm erases some features by smoothing the homogeneous region, which may neglect some information, so for the hyperspectral image data in the same region, simultaneous spectral-based classification and spatial-spectral joint classification for the same region and the classification results are compared and analyzed with certain significance.

The final experimental results show that the semisupervised classification method SADGAN combined with the spectral feature extraction method can achieve excellent classification performance, effectively differentiate geological bodies, and provide an efficient auxiliary means for regional geological mapping.

### Visualization of the results generated by the ${\text{G}}$ Model of the SADGAN

**Figure 11:**
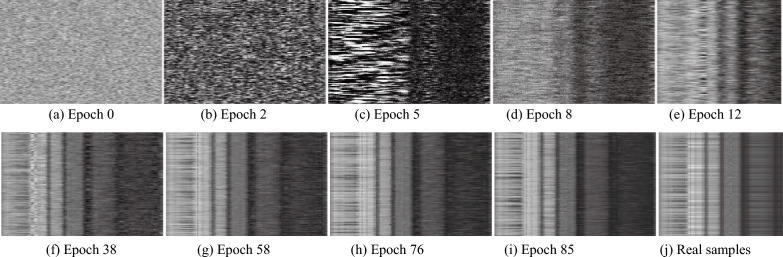
Spectral images (every subplot is an image consisting of 128 lines, and one line is one generated spectrum) generated by $G$ using unlabeled samples after different epochs on Indian Pines. (j) An image of 128 real spectral lines.

**Figure 12:**
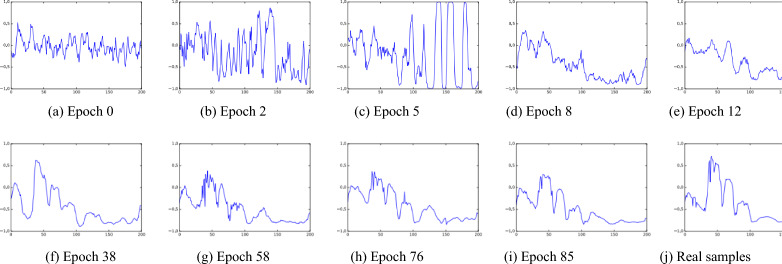
Spectral waveforms generated by $G$ using unlabeled samples after different epochs on the Indian Pines dataset. (j) A real spectral waveform.

In the training step of the SADGAN, the $G$ model will generate spectral samples in each epoch. The results generated by $G$ are visualized in Figs. 11 and 12 for the Indian Pines image. In Fig. 11, every subplot is an image consisting of 128 lines that represents spectral samples with 200 bands. Figure 11a–i displays the images comprising the “fake” spectra generated by $G$, and the subtitle indicates the numerical order of the training epochs in Algorithm 1. Figure 11 (j) shows real spectra. A set of spectral curves generated by $G$ are shown in Fig. 12a–i, which show to the “fake” spectral curves generated by $G$, and the subtitle indicates the numerical order of the training epochs. Figure 12 (j) is a real spectral curve sample. Figures 11 and 12 show that as the number of epochs increases, the curve generated by the generator gradually becomes more similar to the real curve.

### Effect of different size of unlabeled data

The number of unlabeled samples is an important parameter in the SADGAN and can affect the training time of the SADGAN and the accuracy of the final classification. We conducted an experiment to show the effect of the SADGAN on a different number of unlabeled samples. In the CNN training phase, the training data are 10% of the Indian Pines dataset. In Table 8, the first row shows the unlabeled-to-total sample ratio, and the second shows that the SADGAN training time is directly related to the number of samples. With a reduction in unlabeled data, the training time is reduced. From the third row, we can see that as the number of samples decreases, the accuracy decreases. When we use 10% of the total data (unlabeled) to train the SADGAN, the accuracy of the $D$ model after CNN training approaches that of the 1DCNN (in Table 2).

**Table 8:** Effect of different amounts of unlabeled data for the Indian pines dataset.

Unlabeled-to-total sample ratio	100%	50%	25%	10%
Time (s) of training of SADGAN	898	492	260	158
Overall accuracy (OA)	88.6	83.3	78.9	78.6

### Experiment with the spectral angle distance loss function

**Figure 13:**
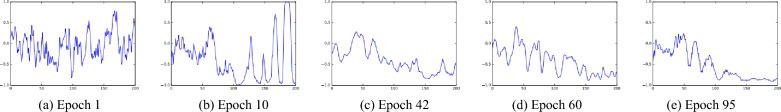
Spectral waveforms generated by $G$ using a binary loss function after different epochs on the Indian Pines dataset.

**Figure 14:**
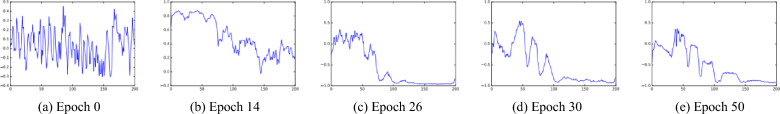
Spectral waveforms generated by $G$ using the proposed spectral angle distance loss function after different epochs on the Indian Pines dataset.

We performed an experiment to compare the results produced by generator $G$ using spectral angle distance loss functions with binary loss functions during the training phase. Traditional GANs use the binary loss function to train themselves. In our experiment, the spectral angle distance is used as the loss function of the $G$ model to accelerate GAN training. Figure 13 shows the results generated by $G$ with the binary loss function. Figure 14 shows the resultant spectral angle distance loss function, where the subtitle indicates the numerical order of the training epochs. From the number of epochs, we can see that the generator $G$ with a spectral angle loss function can produce a more realistic spectrum and perform better.

**Figure 15:**
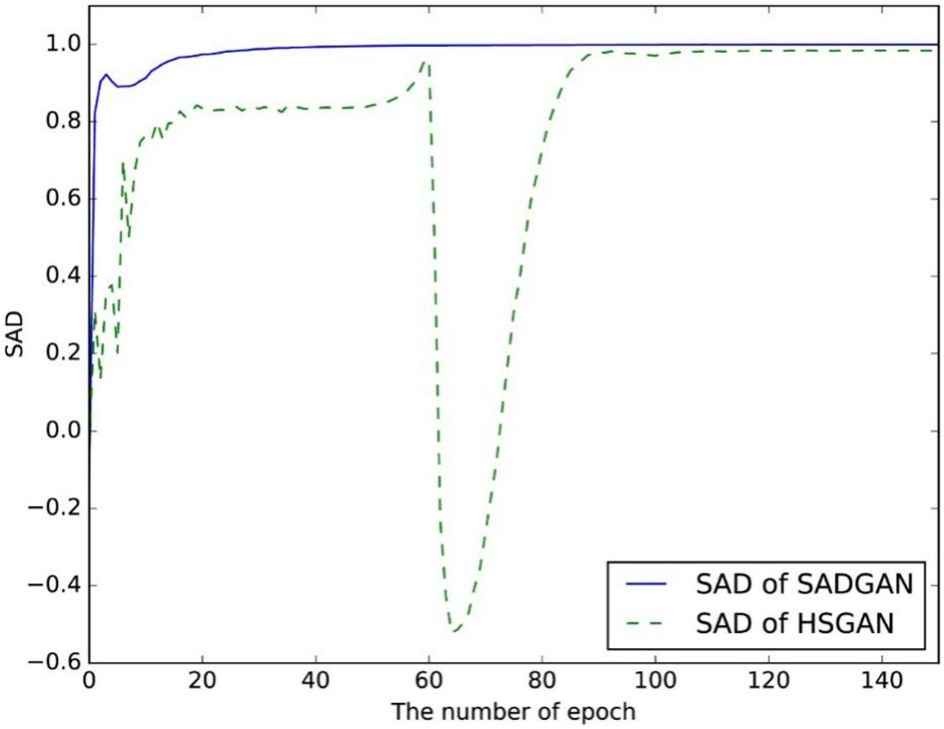
Spectral angle distances of the HSGAN and SADGAN with different epochs.

Figure 15 shows the values of the SAD between the real spectral curve and those generated by the SADGAN and HSGAN using binary loss functions. The training data used in the two methods are from the sixth class (grass-trees, 730 samples) in the Indian Pines dataset. According to Eq. (7), we set the average of the samples generated by the $G$ model in the HSGAN and SADGAN as $a$ and the average of the real sixth class as $b$. The experiment is performed to find the SAD value between ${\varvec{a}}$ and ${\varvec{b}}$ in each iteration. It can be seen from Fig. 13 that as the iteration proceeds, the SAD value of the SADGAN method with the SAD loss function can quickly and stably generate a spectral curve close to the real data (a SAD value close to 1 indicates that the curves are similar). Because of the instability in the training phase of the GAN^23^, the curve of the HSGAN’s SAD suddenly drops between 60 and 90. In comparison, the curve of the SAD of the SADGAN is very smooth, which indicates that our method is more stable during training.

## Conclusion

We proposed a novel semisupervised HSI classification algorithm (SADGAN) by introducing spectral angle distance as a loss function and multilayer feature fusion in the GAN. Traditional GANs are designed to generate 2D natural images without considering the characteristics of the spectrum itself. The spectral angle mapper is an important spectral matching method. Using the spectral angle distance as the loss function of the GAN, the convergence of $G$ is accelerated, and the GAN can generate samples closer to the real spectrum. When the GAN model is trained using all unlabeled samples, the discriminator will acquire the ability to extract the features of all samples. Using the multilayer features of the discriminator and a few labeled samples, we can train a HIS classifier. The proposed method was validated on four hyperspectral datasets and was proven to outperform state-of-the-art methods. To show the effect of the SADGAN more intuitively, the results of a generative model for HSI are visualized and analyzed. By comparing the SAD values between the samples generated by the generator and the real samples, the proposed method can significantly make GAN training more efficient and stable. Detailed experimental analysis also demonstrates that both the spectral angle distance and multilayer feature fusion play important roles in improving the classification performance.

## Data Availability

The data in this paper are available from the Grupo de Inteligencia Computacional (GIC) website (http://www.ehu.eus/ccwintco/index.php/Hyperspectral_Remote_Sensing_Scenes).
